# Acute abdomen caused by torsion of the omentum: A pediatric case report

**DOI:** 10.1097/MD.0000000000029184

**Published:** 2022-04-15

**Authors:** Yue Wang, Ran Huang, Chun Li, Weisong Li

**Affiliations:** Department of Pediatric Surgery, The First Affiliated Hospital of Anhui Medical University, 218 Jixi Road, Shushan District, Hefei City, Anhui Province, China.

**Keywords:** acute abdomen, case report, laparoscopic, omentum, pediatric, torsion

## Abstract

**Rationale:**

Torsion of the omentum and infarction are rare and unusual disorders that often present as acute abdominal pain in the population. The diagnosis of omental torsion is based on clinical and imaging examinations.

**Patient concerns:**

A 7-year-old girl presented with acute right lower quadrant abdominal pain, with symptoms resembling acute appendicitis.

**Diagnosis:**

The patient was diagnosed with omental torsion based on imaging and laparoscopy.

**Interventions:**

Laparoscopic exploration was performed.

**Outcomes:**

The patient was discharged seven days after satisfactory postoperative recovery.

**Lessons:**

Omental torsion should be included in the differential diagnosis of acute abdominal pain, particularly in patients with free hemorrhagic fluid in the abdominal cavity and pelvis.

## 1 Introduction

Torsion of the omentum and infarction are rare and unusual disorders that often present as acute abdominal pain. More than half of the cases (83 cases, 52.5%) were in the age group of 30 to 50 years, and the number of male patients was twice that of female patients.^[1]^ To date, there have been very few reports on torsion of the omentum in children. Because the clinical signs and symptoms of omental torsion are similar to those of other common acute conditions, it is difficult to make a correct preoperative diagnosis.^[2]^ Most cases are diagnosed intraoperatively; however, the combined use of computed tomography (CT) and ultrasound makes preoperative diagnosis possible. Herein, we present a case of laparoscopic omentectomy and appendectomy for primary omental torsion in a child with acute appendicitis that could not be ruled out.

## 2 Case presentation

**Figure 1:**
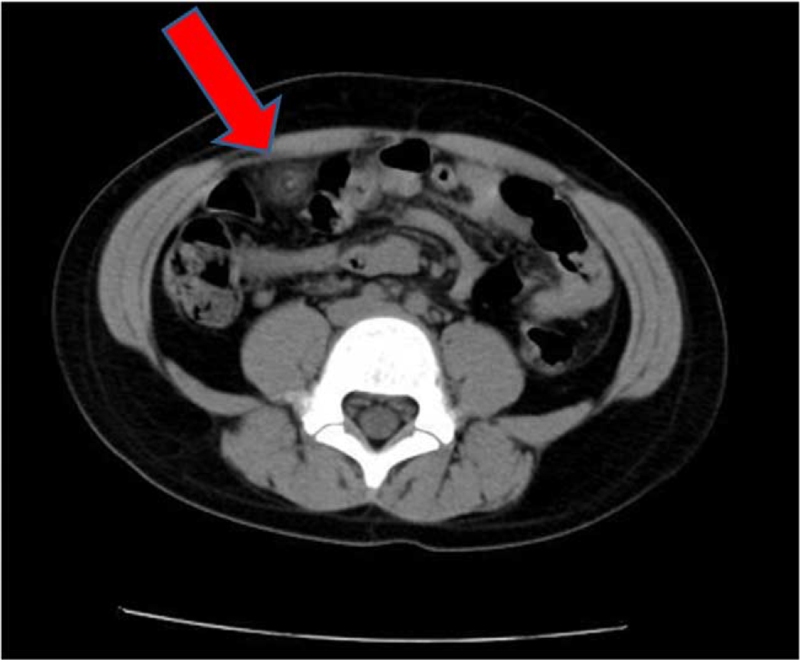
CT scan images of the omental torsion with concentric lines of fat and fibrous tissue surrounding the high density of central vessel at the right lower quadrant and iliac fossa (red arrow).

A 7-year-old Chinese girl presented to the Department of Pediatric Surgery, with acute right lower quadrant abdominal pain lasting for close to 2 days duration. She did not complain of symptoms, such as fever, nausea, vomiting, or diarrhea, but she had a history of occasional abdominal pain. She had no previous abdominal surgery or evidence of recent infection. Heart and lung examinations were unremarkable. Physical examination revealed a temperature of 37.1°C, pulse rate of 98/min, respiratory rate of 23/min, blood pressure of 104/62 mm Hg, height of 130 cm, weight of 32 kg, and body mass index was 18.93 kg/m^2^. Abdominal examination revealed tenderness in the right lower quadrant without rebound tenderness or muscle tension, while the bowel sounds were normal. Laboratory tests demonstrated leukocytosis (11.68 × 10^9^/L), normal percentage of neutrophils, and an elevated hs-C-reactive protein (69.06 mg/L). Abdominal ultrasonography showed effusion in the right lower ventral space and no apparent enlargement of the appendix. CT was performed because physical examination, laboratory tests, and abdominal ultrasound could not confirm the cause of the abdominal pain. A CT scan of the abdominal and pelvic cavity revealed concentric lines of fat and fibrous tissue surrounding the high-density central vessel in the right lower quadrant and iliac fossa (Fig. 1).

**Figure 2:**
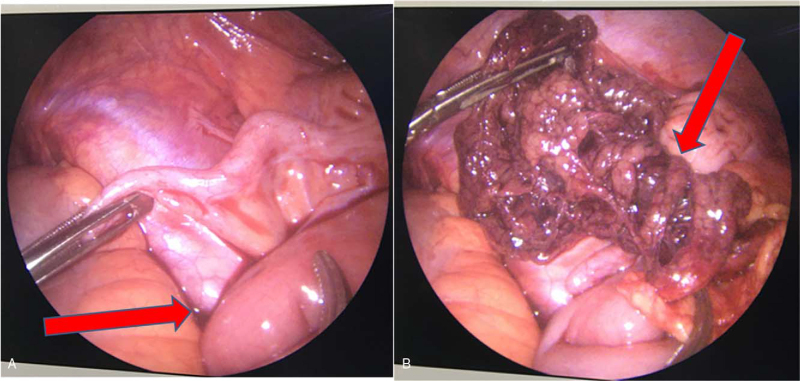
(A) Intraoperative view of free intra-abdominal hemorrhagic fluid; (B) Right part of her omentum was twisted around its vascular axis several times.

**Figure 3:**
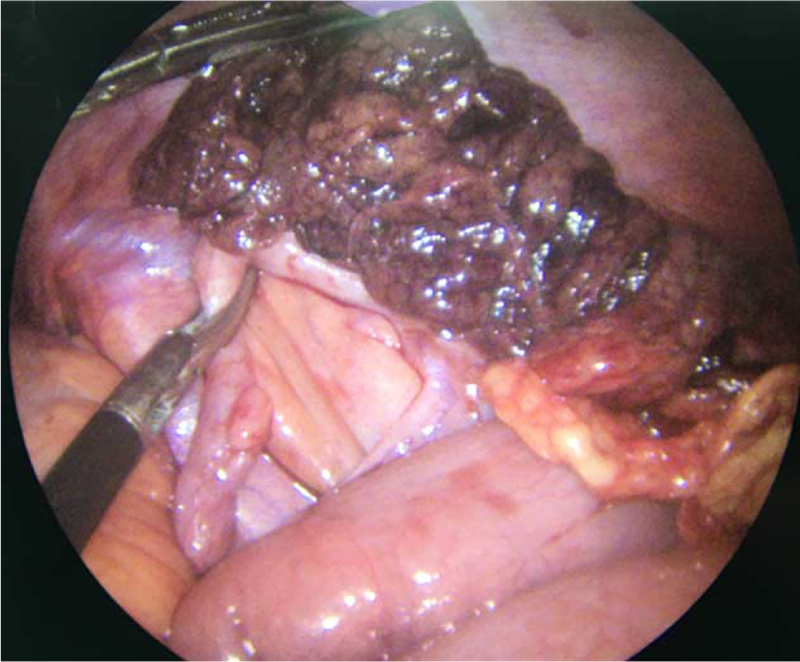
Necrotic segment of her omentum and there was no apparent enlargement of the appendix.

A diagnosis of omental torsion was made, and acute appendicitis could not be excluded; therefore, laparoscopic exploration was performed. The exploration was performed using 3 laparoscopic ports. Initially, the first 10-mm trocar was placed in the upper part of the umbilicus. Pneumoperitoneum was achieved at a pressure of 10 mm Hg using carbon dioxide. Two accessory trocars (5 mm each) were inserted under direct vision in the left lower quadrant and left central quadrant. The procedure was performed using standard laparoscopic instruments and a 30° 5-mm telescope. During the abdominal exploration, free hemorrhagic fluid was first observed in the abdominal and pelvic cavities (Fig. 2A). Further exploration revealed that the right part of her omentum was twisted around its vascular axis several times, as shown in Fig. 2B. There were no obvious abnormalities in the other contents of the abdominal and pelvic cavities. During the surgery, we communicated the condition with the patient's parents. To prevent the onset of appendicitis in the future, the patient's parents decided to remove the appendix at the same time. Because of the necrotic segment of the omentum (Fig. 3), partial omentectomy and appendicectomy were performed. The necrotic omentum was secured 3 cm above the actual torsion using Hem-o-lok clips and was resected. The resected portion was retrieved using a bag via the umbilical port. The surgery was successful, and the patient returned to the ward safely.

**Figure 4:**
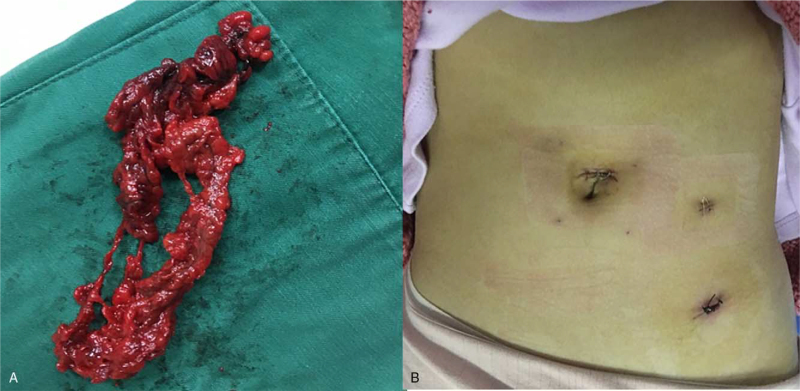
(A) Macroscopic findings. The 7 cm × 5 cm-sized specimen is twisted and dark red in color, suggesting infarction; (B) The patient recovered well after surgery without any complications, and she was discharged from our hospital 7 d later.

Macroscopically, the postoperative specimen showed a size of about 7 cm × 5 cm, suggesting infarction according to twisting and dark red in color (Fig. 4A). Histologically, microscopic examination revealed the specimen to be omental tissue, accompanied by extensive congestion, hemorrhage, and partial necrosis. Appendix showed early acute simple appendicitis.

She recovered well after surgery without any complications and was discharged from our hospital 7 days later (Fig. 4B).

## 3 Discussion

Torsion of the omentum is a rare clinical disorder characterized by twisting of the omentum along its long axis that ultimately leads to vascular impairment. It is an uncommon cause of acute abdominal pain, accounting for approximately 1.1% of all acute abdominal cases.^[3]^ When compared with appendicitis, the incidence of this pathology is 0.0016% to 0.37%, accounting for less than 4 in 1000 acute appendixes.^[4]^ Preoperative diagnosis of omental torsion is difficult, and only 0.6% to 4.8% of cases are accurately diagnosed before operation.^[5]^ Particularly in pediatric cases, the incidence of omental torsion was estimated to be between 0.024% and 0.1% in patients undergoing surgery for suspected acute appendicitis.^[6,7]^

Torsion of the omentum can be divided into primary and secondary, with the latter having a higher incidence.^[4]^ Primary omental torsion is a rare condition that occurs without an associated or secondary intraperitoneal pathology.^[8]^ The first report of omental torsion with no underlying pathology in the literature was attributed to Eitel in 1899.^[3]^ The pathogenesis of primary omental torsion is unclear, but it has several “predisposing factors”, such as excessive fat deposition (obesity) and anatomical abnormalities of the omentum.^[3]^ Precipitation factors include strenuous exercise, sudden changes in body position, gastrointestinal motility after satiety, cough, sneezing, inflammation, edema, and trauma.^[3,9]^ Secondary torsion is more common and has the following underlying causative factors: omentum bifid, inguinal hernia, tumors, cysts, inflammation, previous abdominal surgery, and trauma.^[10]^ Because the anatomic cause or pathology could not be determined in our patient, a diagnosis of primary omental torsion was made.

The clinical presentations of the primary and secondary omental torsion were nearly identical. There were no characteristic signs or symptoms in either primary or secondary omental torsion. The predominant symptom of omental torsion is worsening nonradiating abdominal pain, mostly localized in the right lower quadrant, while fever is not a common presentation. Torsion of the left omentum is very rare because it is shorter, lighter, and less mobile than that of the right side. Gastrointestinal symptoms such as nausea, vomiting, and diarrhea are less common.^[11]^ In addition, on physical examination, abdominal tenderness, rebound tenderness, and muscle tension are more common in the right lower quadrant, and abdominal masses can sometimes be palpable.^[12]^ Omental torsion can mimic a variety of other acute abdominal conditions such as acute appendicitis, acute cholecystitis, acute pancreatitis, digestive tract perforation, intestinal obstruction, and gynecologic diseases.^[13]^ In children, the differential diagnosis may also include acute intussusception, mesenteric lymphadenitis, allergic purpura, and ascariasis. Therefore, it is difficult to accurately diagnose omental torsion preoperatively. Laboratory tests often suggest leukocytosis, elevated C-reactive protein levels, and an elevated erythrocyte sedimentation rate.^[14]^ Laboratory test results are also nonspecific and cannot differentiate other abdominal disorders. Therefore, relying on laboratory test results may delay diagnosis and lead to an increase in the degree and severity of omental torsion.

Most cases are diagnosed intraoperatively; however, the combined use of CT and ultrasound makes preoperative diagnosis possible.^[6,15]^ A CT scan shows either a whirling pattern of fibrous and fatty folds within the omentum or an inflamed fat-containing mass with concentric streaks around the vascular pedicle. The key to diagnosing omental torsion is the presence of a characteristic concentric linear strand. Ultrasonography revealed a hyperechoic, incompressible, oval mass close to the abdominal wall, often with free intra-abdominal fluid.^[16]^ Hence, ultrasonography is also essential to confirm the diagnosis of omental torsion preoperatively, in the absence of other abdominal pathological signs. On the basis of the patient's characteristic CT findings, the preoperative diagnosis was consistent with the final surgical exploration.

Under different conditions, both conservative and surgical treatments can be used for primary omental torsion.^[17]^ Generally, conservative treatment includes prophylactic antibiotics and analgesics, although select patients should undergo careful imaging to rule out other acute abdominal pathologies. Complications such as abscess formation, adhesions, and intestinal obstruction may occur during conservative treatment due to the persistent presence of necrotic tissue in the omentum.^[18]^ However, there have been cases of primary omental torsion successfully cured with conservative treatment.^[19]^ Surgical treatment should be actively selected when the diagnosis is uncertain or the patient's clinical, laboratory, and radiological manifestations worsen with conservative treatment. Surgical resection of the affected omentum, possibly achieved laparoscopically, is the best treatment option.^[20]^ The advantages of laparoscopic techniques include a thorough examination of the abdominal cavity to confirm diagnosis, facilitated aspiration of exudate, and peritoneal irrigation, in addition to the benefits of minimally invasive surgery, which include decreased postoperative pain and wound-related complications.^[21]^ The pathophysiology of omental torsion involves twisting around the vascular axis several times, ultimately resulting in vascular damage and compromised blood supply. As the degree of torsion increases, the arteries gradually occlude, leading to acute hemorrhagic infarction and eventually to necrosis of the omentum. Therefore, to avoid a large amount of toxins returning to the blood through the omentum vein and aggravating the symptoms of postoperative poisoning, the necrotic omentum cannot be recovered before resection.

In conclusion, primary torsion of the omentum is a rare pathology with acute abdomen as the main manifestation, and the majority of cases are misdiagnosed as acute appendicitis. The concentric lines of fat and fibrous tissue often seen on CT scans may prompt preoperative diagnosis and determine the surgical approach. The laparoscopic approach allows thorough exploration of the abdominal cavity and further surgical intervention, especially in patients with ambiguous symptoms and nonspecific abdominal pain. The possibility of omental torsion should be considered when free hemorrhagic fluid is present in the abdominal cavity.

## Acknowledgments

The authors thank the patient and his family for their consent to publish this report.

## Author contributions

**Data curation:** Ran Huang.

**Supervision:** Ran Huang, Chun Li, Weisong Li.

**Visualization:** Chun Li, Weisong Li.

**Writing – original draft:** Yue Wang, Weisong Li.

**Writing – review & editing:** Yue Wang.
